# Determining the reasons for unmet healthcare needs in South Korea: a secondary data analysis

**DOI:** 10.1186/s12955-021-01737-5

**Published:** 2021-03-20

**Authors:** Boyoung Jung, In-Hyuk Ha

**Affiliations:** 1 Department of Health Administration, Hanyang Women’s University, 200 Salgoji-gil, Seongdong-gu, Seoul, 04763 Republic of Korea; 2 Jaseng Spine and Joint Research Institute, Jaseng Medical Foundation, 3F, 538 Gangnam-daero, Gangnam-gu, Seoul, 06110 Republic of Korea

**Keywords:** Unmet healthcare needs, Korean National Health and Nutrition Examination Survey, Anderson’s Behavioral Model of Health Services Use, Socioeconomic status

## Abstract

**Background:**

“Unmet healthcare needs” refers to the situation in which patients or citizens cannot fulfill their medical needs, likely due to socioeconomic reasons. The purpose of this study was to analyze factors related to unmet healthcare needs among South Korean adults.

**Methods:**

We used a retrospective cross-sectional study design. This nationwide-based study included the data of 26,598 participants aged 19 years and older, which were obtained from the 2013–2017 Korea National Health and Nutrition Examination Surveys. Using multiple logistic regression models, we analyzed the associations between factors that influence unmet healthcare needs and participants’ subgroups.

**Results:**

Despite South Korea’s universal health insurance system, in 2017, 9.5% of South Koreans experienced unmet healthcare needs. In both the male and female groups, younger people (age 19–39) had a higher odds ratio (OR) of experiencing unmet healthcare needs compared to older people (reference: age ≥ 60) (men: OR 1.83, 95% confidence interval [CI] = 1.35–2.48; women: OR 1.42, 95% CI 1.12–1.81). In particular, unlike men, women’s unmet healthcare needs increased as their incomes decreased (1 quartile OR 1.55, 2 quartiles OR 1.29, 3 quartiles OR 1.26). Men and women showed a tendency to have more unmet healthcare needs with less exercise, worse subjective health state, worse pain, and a higher degree of depression.

**Conclusions:**

The contributing factors of unmet healthcare needs included having a low socioeconomic status, high stress, severe pain, and severe depression. Considering our findings, we suggest improving healthcare access for those with low socioeconomic status.

**Supplementary Information:**

The online version contains supplementary material available at 10.1186/s12955-021-01737-5.

## Background

Developing and updating policies related to healthcare access are important objectives for improving healthcare equity in Organization for Economic Cooperation and Development (OECD) countries. Though healthcare systems vary in access to services, public health information can help improve the equity of health policies and affect decision-making [1–3]. According to the 2000 World Health Report, published by the World Health Organization (WHO) [4], a healthcare system is a means of improving health that ensures access to care based on needs, not on ability to pay. In order to examine this, it is important to consider “unmet healthcare needs,” which are indicators used globally to assess healthcare accessibility [5, 6].

The definition of an “unmet need” varies among researchers [7]. However, according to the European parliament, an “unmet healthcare need” is a situation in which no satisfactory method of prevention, diagnosis, and treatment exist [8]. Between 2016 and 2017, the rates of unmet healthcare needs across 27 European countries declined from 2.6% to 1% [9]. Multiple organizations, such as the Korea National Health and Nutrition Examination Survey (KNHANES), the Community Health Survey (CHS), the Korea Health Panel Survey, and the Korean Welfare Panel Study, have performed secondary data analyses of “unmet healthcare needs” to determine the healthcare status in South Korea. This refers to a situation in which patients or citizens cannot fulfill their medical needs, most likely due to socioeconomic reasons. Notably, KNHANES reported that the rate of unmet healthcare needs in South Korea is steadily declining, falling from 22% in 2007 to 8.8% in 2017 [10].

Most previous studies of unmet healthcare needs are limited in that their data only includes information from one year [11–13] or only targets certain groups of participants (e.g., certain age groups [14–16], women [17], low-income individuals [13, 18, 19], or people with disabilities [20]). However, to integrate different perspectives and opinions of unmet needs, it is crucial to identify and understand the determinants of such needs [21]. According to Chen and Hou [22], there are three main causes of unmet healthcare needs: (a) availability, which is influenced by factors such as long wait times and shortages of services; (b) accessibility, which includes financial and transportation barriers; and (c) acceptability, which relates to patients who are too busy to seek care or who ignore their health problems). Previous studies [11–19] have indicated that most of the reasons for unmet healthcare needs were economic-related; however, a recent study [10] shows that other reasons surpassed the economic reasons.

The purpose of this study was to analyze the socioeconomic factors related to unmet healthcare needs to recommend effective policies that can address this overall issue of healthcare needs. Thus, we applied a multi-dimensional approach (considering how associated factors affect unmet healthcare needs and stratifying the sex and age) to examine the effects of unmet healthcare needs among adults aged 19 years and older.

## Methods

### Data source

**Fig. 1:**
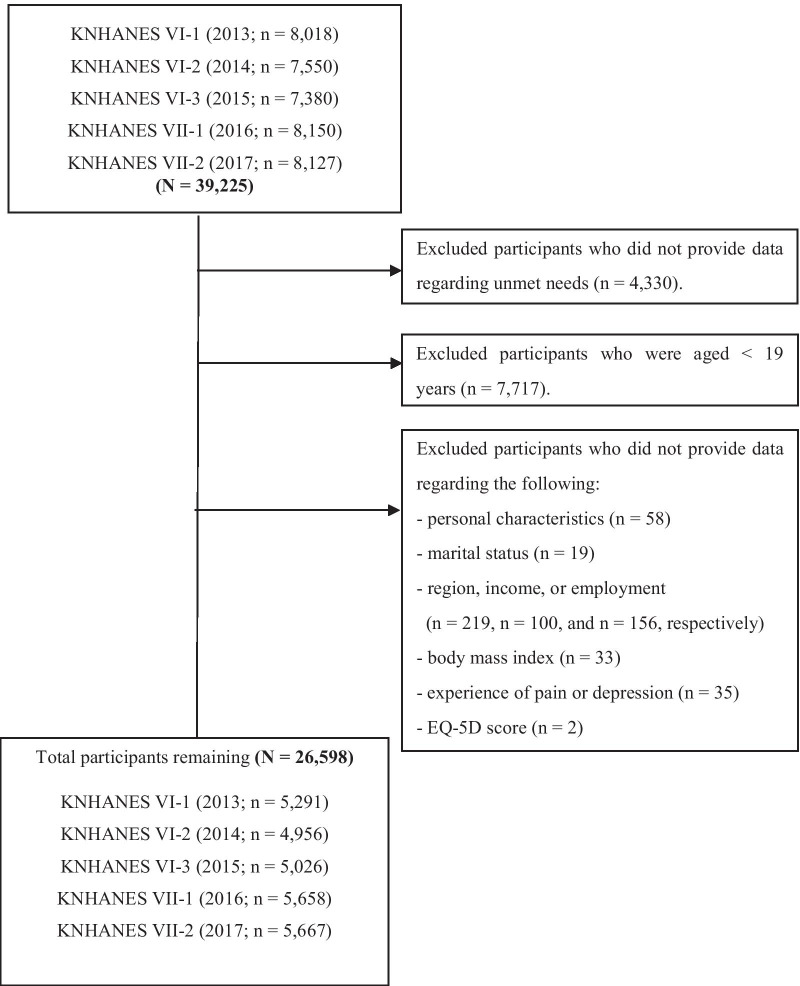
Flow diagram of participant selection

We analyzed data collected by KNHANES, which were originally sourced via three different methods: health-focused interviews, nutrition surveys, and health screenings. Since 1998, KNHANES has collected general population data concerning several indicators, including general health, health behaviors, and socio-demographic characteristics [14]. In the present study, we used data from 2013 to 2017 (waves VI and VII), which provided data for 26,598 adults (11,366 men and 15,232 women) aged ≥ 19 years. Individuals who did not respond to relevant items and those who provided invalid responses were excluded (Fig. 1).

### Outcomes and other variables

#### Dependent variable

For our analysis, we set the dependent variable as whether a respondent had experienced unmet healthcare needs. The reasons for unmet healthcare needs were then divided into three subcategories (“economic,” “time,” and “other”), adopted from Chen and Hou [22]. Overall, the presence of unmet healthcare needs was measured by the question: “Over the past year, have you ever felt that you could not or did not access a medical service at the time when you needed it?” Respondents answered “yes” or “no.” Those who answered “yes” to the question were then asked to provide the reason: “What was the reason for which you did not receive the medical service you needed?” It is crucial to recognize the causes of unmet healthcare needs to achieve a holistic perspective of this matter [22, 23].

Economic reasons meant that the necessary service was not provided for economic reasons. Time reasons meant that the necessary service was not obtained owing to time-related aspects. Others include a variety of reasons, such as “mild symptoms,” “traffic,” “long waiting periods,” “difficulty in scheduling appointments,” “fear of treatment,” “and so on” (Table 1).

**Table 1:** Classification of self-reported unmet healthcare needs from the KNHANES 2013–2017

Year	Stated reasons for unmet healthcare needs						Total
	Economic		Time		Other		
	n	%	n	%	n	%	
2013	205	29.50	217	31.22	273	39.28	695
2014	165	27.27	206	34.05	234	38.68	605
2015	165	25.70	211	32.87	266	41.43	642
2016	116	22.35	232	44.70	171	32.95	519
2017	91	17.14	251	47.27	189	35.59	531
Total	742	24.80	1117	37.33	1133	37.87	2992

#### Predictor variables

We used Anderson’s Behavioral Model of Health Services Use to determine the risk factors that lead to unmet healthcare needs [24, 25]. This model is a framework designed to elucidate determinants associated with the use of health services, and it has been widely utilized in health-service-related research. The factors presented in Anderson’s model are classified into three categories.

*(1) Predisposing factors* These are basic personal characteristics that are largely unrelated to medical needs. Of these, this study included the following: sex (man/woman), age (19–39, 40–59, ≥ 60 years) [16], marital status (married and cohabiting; married and not cohabiting, bereaved, or divorced; unmarried), family type (solo, first generation, second generation, third generation or higher), and education level (elementary school or lower, middle school, high school, college or higher).

*(2) Enabling factors* These factors refer to the resources available to individuals and communities that facilitate access to medical services. Of these, this study included region (Seoul, metropolitan, or rural areas) [26, 27], employment status (“yes” or “no”), occupation type (“white collar,” “pink collar,” “blue collar,” or “unemployed or other”), income (i.e., income quartile; 4Q–1Q), health insurance type (“National Health Insurance [NHI],” “Medicaid,” or “no/do not know;” in South Korea, Medicaid is a type of health insurance funded by the federal and local government that provides health coverage for people with low income) [28], and whether the respondent had private insurance (“yes,” “no,” or “do not know”) [29]. By examining the enabling factors concerning region, employment type, income, and others, the uneven distribution of medical resources, which has been identified as a major challenge in South Korea, could be analyzed [27].

*(3) Need factors* These are associated with disabilities or behaviors that are directly related to the use of healthcare. We included smoking history (three groups: “current smoker,” “past smoker,” and “non-smoker”), alcohol consumption (“never drink,” “less than once per month,” “1–4 times per month,” and “ ≥ 5 times per month”), body mass index (“underweight,” “normal weight,” and “obese”), exercise level (“none,” “mild,” and “high”), self-rated health status (“very good,” “good,” “fair,” “poor,” and “very poor”), stress level (“high,” “moderate,” “low,” and “none”), pain (“none,” “mild,” and “severe”), and depression (“none,” “mild,” and “severe”).

### Statistical analysis

The KNHANES is based on a complex sample design; therefore, all data were analyzed through complex sample analysis, considering weights, stratification variables, and colony variables. A cross-tabulation (chi-square test of independence; χ^2^ test) of the complex sample analysis results (using various characteristics of the study respondents) was performed to identify generally perceived unmet healthcare needs. Using χ^2^ tests, categorical variables were presented as proportions (n, %), while continuous variables were expressed as estimate ± standard error (SE), using a linear model. In addition, risk factors related to unmet healthcare needs were analyzed using χ^2^ tests.

Multiple logistic regression analyses were performed after adjusting for predisposing, enabling, and need factors. Additionally, all analyses were stratified by sex and age (19–39 years/40–59 years/ ≥ 60 years) to identify differences between sex and age regarding unmet healthcare needs. The equations of the logistic regression analyses are below, where $p_{i}$ is the probability that each individual i develops dementia:

Model 1 $$F_{0i} = \log \frac{{p_{i} }}{{1 - p_{i} }} = \beta_{0i} + \beta_{1i} Sex_{i} + \beta_{2i} Age_{i} + \beta_{3i} Marital\;status_{i} + \beta_{4i} Family\;member _{i} + \beta_{5i} Education\;level_{i} \ldots$$

Model 2 $$F_{1i} = F_{0i} + \beta_{6i} Region_{i} + \beta_{7i} Employment_{i} + \beta_{8i} Income_{i} + \beta_{9i} Occuption_{i} + \beta_{10i} Medical\;insurance\;type_{i} + \beta_{10i} Private\;insurance_{i} \ldots$$

Model 3 $$F_{2i} = F_{1i} + \beta_{11i} Smoking\;history_{i} + \beta_{12i} Alcohol\;consumption_{i} + \beta_{13i} Body\;mass\; index + \beta_{14i} Exercise_{i} + \beta_{15i} Self\;rated\;health\;status_{i} + \beta_{16i} Stress\;level_{i} + \beta_{17i} Depression _{i} \ldots$$

The discriminatory power of the models was analyzed using a receiver operating characteristic curve; the area under the curve (AUC) was used to determine the model fit (the closer this value is to 1, the better the model fit). All statistical analyses were performed using SPSS version 25.0 (SPSS Inc., Chicago, IL, USA) and SAS version 9.4 (SAS Institute Inc, Cary, NC), and significance was set at p < 0.05.

### Ethics statement

KNHANES waves VI and VII were conducted by the Korea Center for Disease Control and Prevention (KCDC). All survey protocols were approved by the institutional review board of the KCDC (nos: 2013-07CON-03-4C, 2013-12EXP-03-5C, and 2015-01-02-6C). Informed written consent was obtained from all participants prior to administering the KNHANES, which was conducted in accordance with the Declaration of Helsinki. The original data are publicly available free of charge from the KNHANES website (http://knhanes.cdc.go.kr) for the purposes of academic research. Due to the retrospective nature of this study, which utilized data with encrypted personal information, it was exempted from ethical approval in writing by the Institutional Review Board of Jaseng Hospital of Korean Medicine in Seoul, South Korea (no. 2019-08-001). All authors read and followed the tenets of the Declaration of Helsinki in preparing this study.

## Results

A total of 26,598 adults participated in this study. After weighting was applied, the results represented an estimated 34,997,059 people. Of the 18,216,345 men represented, 1,530,845 (8.4%) reported having had unmet healthcare needs in the past year. Of the 18,942,760 women, 2,545,026 (13.4%) reported experiencing unmet healthcare needs in the past year.

Table 2 illustrates respondents’ general characteristics. In particular, it shows the prevalence of unmet healthcare needs concerning the three factor types (predisposing, enabling, and need).

**Table 2:** Sociodemographic characteristics of the study population by sex (KNHANES 2013–2017)

	Men						Women					
	Total	No		Yes		*p*†	Total	No		Yes		*p*†
	N	n	(%)^a^	n	(%)^a^		N	n	(%)^a^	n	(%)^a^	
Total	18,216,345	16,685,501	91.6	1,530,845	8.4		18,942,760	16,397,734	86.6	2,545,026	13.4	
Age (years)												
19–39	7,134,247	6,454,879	90.5	679,368	9.5	.002	6,729,728	5,815,876	86.4	913,851	13.6	< .001
40–59	7,397,520	6,797,499	91.9	600,021	8.1		7,574,482	6,690,238	88.3	884,244	11.7	
≥ 60	3,684,579	3,433,123	93.2	251,456	6.8		4,638,551	3,891,620	83.9	746,931	16.1	
Marital status												
Married and cohabiting	12,153,503	11,199,408	92.1	954,095	7.9	.005	12,472,117	10,964,171	87.9	1,507,946	12.1	< .001
Married but not cohabiting, or bereaved or divorced	952,475	834,613	87.6	117,862	12.4		3,038,706	2,488,206	81.9	550,500	18.1	
Unmarried	5,110,367	4,651,479	91.0	458,888	9.0		3,431,937	2,945,357	85.8	486,580	14.2	
Number of family members												
1	1,591,858	1,403,956	88.2	187,902	11.8	.001	1,733,710	1,399,696	80.7	334,014	19.3	< .001
2	4,332,376	4,020,407	92.8	311,969	7.2		4,626,732	4,034,561	87.2	592,171	12.8	
3	5,039,486	4,572,429	90.7	467,057	9.3		5,253,312	4,536,896	86.4	716,416	13.6	
4	5,425,420	4,996,671	92.1	428,749	7.9		5,208,615	4,608,789	88.5	599,826	11.5	
≥ 5	1,827,206	1,692,037	92.6	135,168	7.4		2,120,391	1,817,791	85.7	302,600	14.3	
Family type												
Solo	1,591,858	1,403,956	88.2	187,902	11.8	.002	1,733,710	1,399,696	80.7	334,014	19.3	< .001
1st generation	3,627,903	3,370,651	92.9	257,253	7.1		3,424,477	2,990,585	87.3	433,891	12.7	
2nd generation	11,622,273	10,653,148	91.7	969,125	8.3		11,934,761	10,455,630	87.6	1,479,130	12.4	
3rd generation or higher	1,374,311	1,257,746	91.5	116,565	8.5		1,849,813	1,551,822	83.9	297,991	16.1	
Education level												
Elementary school or lower	1,919,911	1,725,704	89.9	194,208	10.1	.031	4,052,135	3,300,280	81.4	751,855	18.6	< .001
Middle school	1,614,136	1,476,394	91.5	137,743	8.5		1,739,105	1,498,772	86.2	240,333	13.8	
High school	7,092,204	6,459,846	91.1	632,358	8.9		6,544,948	5,762,870	88.1	782,078	11.9	
College or higher	7,590,094	7,023,558	92.5	566,536	7.5		6,606,572	5,835,812	88.3	770,760	11.7	
Region												
Seoul	3,712,026	3,429,116	92.4	282,910	7.6	.212	3,957,342	3,451,044	87.2	506,298	12.8	.574
Metro	4,379,973	4,029,841	92.0	350,132	8.0		4,656,018	4,033,340	86.6	622,678	13.4	
Rural	10,124,346	9,226,543	91.1	897,803	8.9		10,329,400	8,913,350	86.3	1,416,050	13.7	
Employment status												
Unemployed	4,465,236	4,143,690	92.8	321,546	7.2	.022	9,248,403	8,056,557	87.1	1,191,846	12.9	.111
Employed	13,751,109	12,541,811	91.2	1,209,299	8.8		9,694,357	8,341,177	86.0	1,353,180	14.0	
Income^b^												
1Q (lowest)	4,550,538	4,115,607	90.4	434,930	9.6	.017	4,701,072	3,866,590	82.2	834,483	17.8	< .001
2Q	4,548,682	4,153,534	91.3	395,148	8.7		4,738,686	4,088,322	86.3	650,364	13.7	
3Q	4,512,661	4,126,987	91.5	385,674	8.5		4,733,493	4,127,687	87.2	605,805	12.8	
4Q (highest)	4,604,465	4,289,372	93.2	315,093	6.8		4,769,509	4,315,135	90.5	454,374	9.5	
Occupation												
White collar	5,675,473	5,225,634	92.1	449,840	7.9	.026	4,210,948	3,697,355	87.8	513,592	12.2	.001
Pink collar	2,157,717	1,986,487	92.1	171,230	7.9		2,787,396	2,415,371	86.7	372,024	13.3	
Blue collar	5,157,143	4,647,831	90.1	509,312	9.9		2,234,315	1,856,211	83.1	378,104	16.9	
Unemployed or other	5,226,012	4,825,549	92.3	400,463	7.7		9,710,102	8,428,797	86.8	1,281,306	13.2	
Medical Insurance type												
NHI	17,520,008	16,080,991	91.8	1,439,016	8.2	.025	18,075,601	15,743,253	87.1	2,332,347	12.9	< .001
Medicaid	494,275	430,209	87.0	64,066	13.0		646,528	474,806	73.4	171,722	26.6	
No/do not know	202,063	174,300	86.3	27,762	13.7		220,631	179,675	81.4	40,956	18.6	
Private insurance												
Yes	14,384,239	13,223,721	91.9	1,160,517	8.1	.094	15,011,361	13,156,749	87.6	1,854,612	12.4	< .001
No	3,638,702	3,284,231	90.3	354,471	9.7		3,771,901	3,104,828	82.3	667,073	17.7	
Do not know	193,404	177,548	91.8	15,856	8.2		159,498	136,157	85.4	23,341	14.6	
Smoking history												
Non-smoker	4,504,920	4,195,041	93.1	309,879	6.9	< .001	16,636,672	14,478,079	87.0	2,158,593	13.0	< .001
Past smoker	6,460,273	6,014,161	93.1	446,113	6.9		1,120,625	959,229	85.6	161,396	14.4	
Current smoker	7,251,152	6,476,300	89.3	774,853	10.7		1,185,463	960,426	81.0	225,038	19.0	
Alcohol consumption												
Never drink	2,633,850	2,430,482	92.3	203,368	7.7	.544	6,116,013	5,184,505	84.8	931,508	15.2	< .001
Less than 1 time per month	2,040,101	1,872,840	91.8	167,261	8.2		4,465,941	3,957,458	88.6	508,483	11.4	
1–4 times per month	7,012,060	6,386,257	91.1	625,803	8.9		6,068,744	5,279,258	87.0	789,486	13.0	
≥ 5 times per month	6,530,334	5,995,922	91.8	534,412	8.2		2,292,062	1,976,513	86.2	315,549	13.8	
Body mass index												
Normal (18.5 ≤ BMI < 25)	10,540,097	9,671,147	91.8	868,950	8.2	.168	12,549,645	10,972,946	87.4	1,576,699	12.6	.001
Underweight (BMI < 18.5)	510,387	448,310	87.8	62,077	12.2		1,174,258	978,254	83.3	196,005	16.7	
Obese (BMI ≥ 25)	7,165,862	6,566,044	91.6	599,817	8.4		5,218,856	4,446,534	85.2	772,323	14.8	
Exercise												
None	16,706,537	15,445,099	92.4	1,261,438	7.6	< .001	16,274,205	14,414,380	88.6	1,859,825	11.4	< .001
Mild	1,456,613	1,203,825	82.6	252,789	17.4		2,523,305	1,899,396	75.3	623,909	24.7	
High	53,195	36,577	68.8	16,618	31.2		145,250	83,958	57.8	61,292	42.2	
Stress level												
High	4,646,371	3,975,432	85.6	670,939	14.4	< .001	5,380,940	4,288,734	79.7	1,092,207	20.3	< .001
Moderate	10,652,409	9,903,651	93.0	748,758	7.0		10,725,638	9,520,775	88.8	1,204,863	11.2	
Low	2,822,867	2,717,241	96.3	105,626	3.7		2,713,771	2,483,941	91.5	229,830	8.5	
None	94,698	89,177	94.2	5,521	5.8		122,410	104,284	85.2	18,126	14.8	
Self-rated health status												
Very good/good	6,366,031	6,067,433	95.3	298,597	4.7	< .001	5,168,004	4,835,422	93.6	332,582	6.4	< .001
Fair	9,202,108	8,432,601	91.6	769,507	8.4		9,947,397	8,705,500	87.5	1,241,897	12.5	
Poor/very poor	2,648,207	2,185,466	82.5	462,741	17.5		3,827,359	2,856,812	74.6	970,547	25.4	
Pain												
None	15,243,143	14,284,214	93.7	958,930	6.3	< .001	13,975,362	12,620,501	90.3	1,354,861	9.7	< .001
Mild	2,775,714	2,247,400	81.0	528,313	19.0		4,494,769	3,470,452	77.2	1,024,317	22.8	
Severe	197,488	153,887	77.9	43,602	22.1		472,629	306,781	64.9	165,848	35.1	
Depression												
None	16,948,918	15,666,651	92.4	1,282,267	7.6	< .001	16,579,396	14,703,608	88.7	1,875,788	11.3	< .001
Mild	1,202,449	976,826	81.2	225,623	18.8		2,184,118	1,593,660	73.0	590,458	27.0	
Severe	64,978	42,023	64.7	22,955	35.3		179,245	100,466	56.0	78,779	44.0	

A chi-square test was performed to determine the differences between groups with and without unmet needs

*NHI* National Health Insurance, *Q* quartile

^a^Weighted (%)

^b^Income divided by quartile

Concerning sex, women were more likely to experience unmet healthcare needs than men. Within that group, participants aged 60 years and older experienced the highest rate of unmet healthcare needs. For men, the younger age group (19–39 years) experienced the highest rate of unmet healthcare needs as compared to their counterparts. Furthermore, marital status influenced both sexes: singles (separated, widowed, or divorced) experienced more unmet healthcare needs than those who were married. Similarly, for both sexes, single-person families had higher rates of unmet healthcare needs (men: 11.8%; women: 19.3%) as compared to their counterparts. Further, among men and women, those who had the lowest education level (elementary school or below) had the highest levels of unmet healthcare needs (men: 10.1%; women: 18.6%) as compared to their counterparts.

Both men and women from rural areas were more likely to experience unmet healthcare needs when compared to those from other regions (men: 8.9%; women: 13.7%). Regarding women’s income, those with the lowest income showed the highest rate of unmet healthcare needs (17.8%) as compared to their counterparts. By contrast, for men, the rate of unmet healthcare needs did not vary significantly among the income quartile groups. Concerning occupation for both sexes, the blue-collar worker group had the most unmet healthcare needs (men: 9.9%; women: 16.9%) as compared to their counterparts. Regarding health insurance for both sexes, Medicaid beneficiaries had the highest rate when compared to beneficiaries of other types of health insurance (men: 13.0%; women: 26.6%). Finally, women who did not have private insurance had a higher rate of unmet healthcare needs compared to those who had some form of insurance (women: 17.7%).

Regarding need factors, both male and female smokers were more likely to experience unmet healthcare needs (men: 10.7%; women: 19.0%) as compared to their counterparts. In the drinking category, there was no significant difference among the men; however, non-drinking women experienced more unmet healthcare needs (women: 15.2%, p < 0.001) as compared to their counterparts. Body mass index showed no significance among men; however, underweight and obese women experienced more unmet healthcare needs than women who had normal body weight. For both sexes, those who engaged in high levels of exercise and who had high stress levels showed higher rates of unmet healthcare needs as compared to their counterparts. Finally, those who considered themselves to have a poor health status and those who experienced severe pain and depression were more likely to experience unmet healthcare needs as compared to their counterparts.

Table 3 shows the results of the logistic regression model. Model 1 was adjusted by sex, age, marital status, family members, and education level. Model 2 was adjusted by Model 1 as well as region, economic activity, income, occupation, medical insurance type, and private insurance. Model 3 was adjusted by Model 2 as well as smoking, drinking, obesity, exercise, self-rated health status, stress level, pain, and depression. The explanatory power demonstrated improvement in the progression from Model 1 to Model 3 (AUCs of Model 1, Model 2, and Model 3 were 0.600, 0.612, and 0.700, respectively).

**Table 3:** Overall unmet needs according to the analysis model

Variables	Unmet needs, based on KNHANES 2013–2017 data											
	Model 1				Model 2				Model 3			
	OR	95% CI		*p*	OR	95% CI		*p*	OR	95% CI		*p*
Sex												
Male	1.00				1.00				1.00			
Female	1.55	1.41	1.71	< .001	1.72	1.55	1.90	< .001	1.64	1.43	1.87	< .001
Age (years)												
19–39	1.76	1.49	2.08	< .001	1.59	1.33	1.90	< .001	1.60	1.33	1.92	< .001
40–59	1.27	1.11	1.45	.001	1.14	0.98	1.31	.086	1.17	1.01	1.36	.040
≥ 60	1.00				1.00				1.00			
Marital status												
Married and cohabiting	1.03	0.89	1.19	.716	1.02	0.88	1.19	.763	1.00	0.86	1.16	.995
Married but not cohabiting, or bereaved or divorced	1.35	1.11	1.63	.002	1.24	1.03	1.50	.025	1.08	0.89	1.32	.427
Unmarried	1.00				1.00				1.00			
Number of family members												
1	1.29	1.05	1.57	.014	1.17	0.95	1.43	.144	1.06	0.85	1.31	.612
2	0.90	0.76	1.06	.204	0.87	0.74	1.03	.111	0.82	0.69	0.97	.020
3	1.08	0.92	1.28	.345	1.09	0.92	1.29	.322	1.04	0.87	1.24	.663
4	0.92	0.78	1.09	.336	0.94	0.80	1.12	.506	0.93	0.78	1.10	.401
≥ 5	1.00				1.00				1.00			
Education level												
Elementary school or lower	2.08	1.78	2.43	< .001	1.70	1.44	2.02	< .001	1.20	1.00	1.43	.050
Middle school	1.44	1.21	1.72	< .001	1.25	1.04	1.51	.019	1.00	0.82	1.21	.975
High school	1.16	1.03	1.30	.017	1.08	0.95	1.23	.214	1.02	0.90	1.17	.752
College or higher	1.00				1.00				1.00			
Region												
Seoul					1.00				1.00			
Metro					1.04	0.92	1.18	.486	1.07	0.94	1.21	.301
Rural					0.99	0.86	1.15	.904	1.05	0.90	1.22	.541
Employment status												
Employed					1.00				1.00			
Unemployed					1.53	1.23	1.90	< .001	1.74	1.38	2.18	< .001
Income												
1Q (lowest)					1.45	1.26	1.67	< .001	1.29	1.11	1.49	.001
2Q					1.26	1.10	1.45	.001	1.18	1.03	1.36	.020
3Q					1.27	1.10	1.45	.001	1.19	1.03	1.37	.017
4Q (highest)					1.00							
Occupation												
White collar					1.00				1.00			
Pink collar					1.23	0.96	1.57	.106	1.29	1.00	1.66	.052
Blue collar					1.15	0.98	1.35	.087	1.22	1.04	1.43	.015
Unemployed or other					0.95	0.81	1.13	.573	0.95	0.80	1.13	.559
Medical insurance type												
NHI					1.00				1.00			
Medicaid					1.64	1.31	2.06	< .001	1.17	0.77	1.80	.456
No/do not know					1.30	0.85	1.97	.222	1.03	0.81	1.31	.803
Private insurance												
Yes					1.00				1.00			
No					1.19	1.06	1.35	.004	1.14	1.00	1.29	.045
Do not know					0.91	0.57	1.44	.692	1.00	0.61	1.65	.996
Smoking history												
Current smoker									1.26	1.08	1.46	.003
Past smoker									0.94	0.80	1.10	.419
Non-smoker									1.00			
Alcohol consumption												
Never drink									1.00			
Less than one time per month									0.93	0.79	1.08	.327
1–4 times per month									1.05	0.93	1.18	.462
≥ 5 times per month									0.88	0.76	1.01	.069
Body mass index (BMI)												
Normal weight (18.5 ≤ BMI < 25)									1.00			
Under weight (BMI < 18.5)									0.95	0.86	1.06	.374
Obese (BMI ≥ 25)									1.17	0.94	1.47	.162
Exercise												
None									1.00			
Mild									1.96	1.30	2.95	.001
High									1.31	1.13	1.52	< .001
Self-rated health status												
Very poor									3.62	2.46	5.32	< .001
Poor									3.47	2.44	4.95	< .001
Fair									2.25	1.61	3.14	< .001
Good									1.44	1.02	2.04	.038
Very good									1.00			
Stress level												
High									2.35	1.28	4.30	.006
Moderate									1.56	0.85	2.87	.150
Low									1.16	0.63	2.15	.636
None									1.00			
Pain												
None									1.00			
Mild									2.09	1.61	2.72	< .001
Severe									2.06	1.83	2.31	< .001
Depression												
Light									1.00			
Moderate									1.45	1.26	1.65	< .001
Heavy/extreme									1.78	1.23	2.57	.002
AUC^a^	0.600				0.612				0.700			

Logistic regression analysis with a complex sampling design was performed by adjusting for covariates

Model 1 was adjusted for sex, age, marital status, number of family members, and education level

Model 2 was adjusted for Model 1, as well as region, economic activity, income, occupation, medical insurance type, and private insurance

Model 3 was adjusted for Model 1 and Model 2, as well as smoking, drinking, body mass index, exercise, self-rated health status, stress level, pain, and depression

*NHI* National Health Insurance, *Q* quartile, *OR* odds ratio, *CI* confidence interval, *AUC* area under the receiver, *OR* 95%, *CI* 95%

^a^The AUC operating characteristic curve indicates the discrimination ability of the prediction model

Table 4 shows the results of the logistic regression model by sex. In both the male and female groups, younger people (age: 19–39) had a higher odds ratio (OR) of experiencing unmet healthcare needs compared to older people (reference: age ≥ 60) (men: OR 1.83, 95% confidence interval [CI] 1.35–2.48; women: OR 1.42, 95% CI 1.12–1.81). Both groups showed a higher tendency of unmet healthcare needs when the individuals were unemployed (men: OR 1.93, 95% CI 1.38–2.71; women: OR 1.65, 95% CI 1.22–2.25). In particular, unlike men, women’s unmet healthcare needs increased as their incomes decreased (1Q OR 1.55, 2Q OR 1.29, 3Q OR 1.26). Only male smokers showed higher unmet healthcare needs compared to non-smokers (men: OR 1.27, 95% CI 1.02–1.58). Men and women showed a tendency to have more unmet healthcare needs with less exercise, worse subjective health state, worse pain, and a higher degree of depression. The significance of the interaction term was tested with the likelihood test, and if it was significant, each term was analyzed by post-mortem analysis. As a result of the likelihood test, the interaction terms according to all covariates were significant. In particular, the higher the level of education, income, and pain, the higher the odds ratio for unmet medical care for women.

**Table 4:** Overall unmet needs by sex

Variables	Unmet needs, based on KNHANES 2013–2017 data											
	Total				Men				Women			
	OR	95% CI		*p*	OR	95% CI		*p*	OR	95% CI		*p*
Age (years)												
19–39	1.60	1.33	1.92	< .001	1.83	1.35	2.48	< .001	1.42	1.12	1.81	.004
40–59	1.17	1.01	1.36	.040	1.31	1.02	1.67	.036	1.10	0.91	1.34	.334
≥ 60	1.00				1.00				1.00			
Marital status												
Married and cohabiting	1.00	0.86	1.16	.995	1.11	0.86	1.44	.414	0.92	0.76	1.11	.397
Married but not cohabiting, or bereaved or divorced	1.08	0.89	1.32	.427	1.30	0.89	1.90	.172	0.94	0.75	1.18	.594
Unmarried	1.00				1.00				1.00			
Number of family members												
1	1.06	0.85	1.31	.612	1.43	0.98	2.08	.065	0.88	0.69	1.13	.316
2	0.82	0.69	0.97	.020	1.02	0.75	1.40	.889	0.72	0.59	0.89	.002
3	1.04	0.87	1.24	.663	1.33	0.99	1.79	.059	0.91	0.74	1.12	.375
4	0.93	0.78	1.10	.401	1.15	0.85	1.58	.368	0.83	0.68	1.02	.080
≥ 5	1.00				1.00				1.00			
Education level												
Elementary school or lower	1.20	1.00	1.43	.050	1.22	0.89	1.67	.224	1.14	0.91	1.44	.246
Middle school	1.00	0.82	1.21	.975	1.04	0.75	1.44	.820	0.97	0.76	1.24	.807
High school	1.02	0.90	1.17	.752	1.15	0.93	1.43	.195	0.94	0.80	1.11	.442
College or higher	1.00				1.00				1.00			
Region												
Seoul	1.00				1.00				1.00			
Metro	1.07	0.94	1.21	.301	1.13	0.92	1.38	.252	1.02	0.87	1.19	.827
Rural	1.05	0.90	1.22	.541	1.05	0.82	1.33	.718	1.03	0.86	1.23	.751
Employment status												
Employed	1.00				1.00				1.00			
Unemployed	1.74	1.38	2.18	< .001	1.93	1.38	2.71	< .001	1.65	1.22	2.25	.001
Income												
1Q (lowest)	1.29	1.11	1.49	.001	1.00	0.77	1.29	.969	1.55	1.29	1.86	< .001
2Q	1.18	1.03	1.36	.020	1.05	0.83	1.34	.667	1.29	1.09	1.53	.004
3Q	1.19	1.03	1.37	.017	1.11	0.88	1.39	.391	1.26	1.06	1.50	.010
4Q (highest)	1.00				1.00				1.00			
Occupation												
White collar	1.00				1.00				1.00			
Pink collar	1.29	1.00	1.66	.052	1.31	0.91	1.88	.149	1.30	0.92	1.83	.140
Blue collar	1.22	1.04	1.43	.015	1.22	0.97	1.53	.095	1.21	0.96	1.52	.106
Unemployed or other	0.95	0.80	1.13	.559	0.90	0.67	1.20	.468	1.01	0.82	1.25	.906
Medical insurance type												
NHI	1.00				1.00				1.00			
Medicaid	1.17	0.77	1.80	.456	1.54	0.75	3.17	.235	1.16	0.89	1.50	.275
No/do not know	1.03	0.81	1.31	.803	0.90	0.56	1.46	.674	0.97	0.61	1.54	.887
Private insurance												
Yes	1.00				1.00				1.00			
No	1.14	1.00	1.29	.045	1.19	0.95	1.49	.134	1.13	0.97	1.32	.130
Do not know	1.00	0.61	1.65	.996	1.07	0.47	2.45	.868	0.96	0.53	1.75	.892
Smoking history												
Current smoker	1.26	1.08	1.46	.003	1.27	1.02	1.58	.033	1.20	0.96	1.51	.115
Past smoker	0.94	0.80	1.10	.419	0.98	0.77	1.25	.878	0.90	0.71	1.14	.362
Non-smoker	1.00				1.00				1.00			
Alcohol consumption												
Never drink	1.00				1.00				1.00			
Less than once per month	0.93	0.79	1.08	.327	0.99	0.76	1.28	.913	0.96	0.79	1.17	.682
1–4 times per month	1.05	0.93	1.18	.462	1.23	0.95	1.59	.119	0.98	0.85	1.14	.801
≥ 5 times per month	0.88	0.76	1.01	.069	1.04	0.75	1.44	.834	0.83	0.71	0.98	.025
Body mass index (BMI)												
Normal weight (18.5 ≤ BMI < 25)	1.00				1.00				1.00			
Under weight (BMI < 18.5)	0.95	0.86	1.06	.374	0.91	0.77	1.08	.279	0.98	0.86	1.10	.687
Obese (BMI ≥ 25)	1.17	0.94	1.47	.162	1.14	0.70	1.85	.604	1.21	0.94	1.55	.147
Exercise												
None	1.00				1.00				1.00			
Mild	1.31	1.13	1.52	< .001	1.36	1.03	1.80	.029	1.30	1.09	1.55	.004
High	1.96	1.30	2.95	.001	2.95	1.16	7.51	.023	1.78	1.16	2.74	.009
Self-rated health												
Very poor	3.62	2.46	5.32	< .001	3.22	1.62	6.40	.001	3.84	2.37	6.23	< .001
Poor	3.47	2.44	4.95	< .001	3.52	2.00	6.22	< .001	3.52	2.24	5.52	< .001
Fair	2.25	1.61	3.14	< .001	2.19	1.27	3.77	.005	2.30	1.50	3.54	< .001
Good	1.44	1.02	2.04	.038	1.56	0.90	2.73	.116	1.35	0.86	2.13	.188
Very good	1.00				1.00				1.00			
Stress level												
High	2.35	1.28	4.30	.006	2.97	0.78	11.34	.111	2.14	1.08	4.26	.030
Moderate	1.56	0.85	2.87	.150	1.74	0.46	6.62	.414	1.55	0.78	3.09	.213
Low	1.16	0.63	2.15	.636	1.15	0.30	4.51	.837	1.25	0.62	2.52	.534
None	1.00				1.00				1.00			
Pain												
None	1.00				1.00				1.00			
Mild	2.06	1.83	2.31	< .001	1.98	1.02	3.86	.044	1.81	1.57	2.09	< .001
Severe	2.09	1.61	2.72	< .001	2.56	2.09	3.13	< .001	2.04	1.56	2.66	< .001
Depression												
Light	1.00				1.00				1.00			
Moderate	1.45	1.26	1.65	< .001	1.34	1.02	1.76	.039	1.54	1.32	1.80	< .001
Heavy/extreme	1.78	1.23	2.57	.002	2.02	0.92	4.45	.081	1.76	1.16	2.66	.008

Logistic regression analysis with a complex sampling design was performed by adjusting for covariates

The significance of the interaction term was tested with the likelihood test, and if it was significant, each term was analyzed by post-mortem analysis

Model was adjusted for sex, age, marital status, number of family members, education level, region, economic activity, income, occupation, medical insurance type, private insurance, smoking, drinking, body mass index, exercise, self-rated health status, stress level, pain, and depression

*NHI* National Health Insurance, *Q* quartile, *OR* odds ratio, *CI* confidence interval

Table 5 shows the results of the logistic regression model according to age group. Women had higher odds of experiencing unmet healthcare needs compared to men, regardless of age. Young and older adult age groups (19–39 years/40–59 years) showed a tendency to have more unmet healthcare needs when they were unemployed (19–39 years: OR 1.53, 95% CI 1.17–2.01; 40–59 years: OR 2.34, 95% CI 1.63–3.36).

**Table 5:** Overall unmet needs according to age group in the KNHANES 2013–2017

Variables	Unmet needs, KNHANES 2013–2017											
	19–39 years				40–59 years				≥ 60 years			
	OR	95% CI		*p*	OR	95% CI		*p*	OR	95% CI		*p*
Sex												
Male	1.00				1.00				1.00			
Female	1.67	1.25	2.22	.001	1.60	1.25	2.06	< .001	1.55	1.26	1.90	< .001
Marital status												
Married-cohabiting	0.82	0.34	1.97	.651	1.63	1.07	2.50	.024	0.93	0.78	1.11	.426
Married-no cohabiting, bereaved, or divorced	0.84	0.36	1.98	.692	1.71	1.11	2.64	.016	0.62	0.30	1.28	.200
Unmarried	1.00				1.00				1.00			
Number of family members												
1	1.10	0.75	1.62	.616	1.27	0.85	1.91	.239	0.88	0.61	1.28	.504
2	0.87	0.61	1.23	.421	0.85	0.63	1.14	.280	0.74	0.55	0.99	.044
3	0.92	0.63	1.34	.653	1.04	0.78	1.37	.804	1.06	0.82	1.37	.657
4	1.20	0.78	1.83	.410	0.97	0.73	1.30	.854	0.83	0.65	1.07	.145
≥ 5	1.00				1.00				1.00			
Education level												
Elementary school or less	1.74	1.13	2.67	.012	1.09	0.82	1.46	.548	0.87	0.38	2.02	.745
Middle school	1.52	0.95	2.42	.080	0.94	0.72	1.22	.644	0.99	0.56	1.74	.977
High school	1.45	0.92	2.28	.107	1.00	0.82	1.22	.989	1.01	0.84	1.21	.918
College or over	1.00				1.00				1.00			
Region												
Seoul	1.00				1.00				1.00			
Metro	1.00	0.80	1.24	.965	1.04	0.84	1.29	.717	1.14	0.93	1.40	.191
Rural	0.86	0.66	1.12	.256	1.17	0.91	1.50	.215	1.03	0.82	1.30	.794
Employment status												
Employed	1.00				1.00				1.00			
Unemployed	1.53	1.17	2.01	.002	2.34	1.63	3.36	< .001	0.75	0.21	2.70	.657
Income												
1Q (lowest)	1.38	1.06	1.79	.018	1.26	0.99	1.60	.060	1.30	1.01	1.68	.040
2Q	1.19	0.92	1.54	.190	1.05	0.84	1.32	.646	1.31	1.03	1.68	.031
3Q	1.02	0.80	1.32	.850	1.03	0.81	1.31	.787	1.44	1.15	1.81	.002
4Q (highest)	1.00				1.00				1.00			
Occupation												
White collar	1.00				1.00				1.00			
Pink collar	1.06	0.60	1.87	.847	1.88	1.29	2.75	.001	0.56	0.15	2.04	.381
Blue collar	1.27	0.73	2.19	.396	1.36	1.07	1.74	.013	1.08	0.81	1.44	.598
Unemployed or other	1.16	0.64	2.09	.628	1.03	0.79	1.34	.814	0.84	0.65	1.10	.216
Medical insurance type												
NHI	1.00				1.00				1.00			
Medicaid	1.31	0.79	2.18	.291	1.05	0.40	2.75	.919	1.10	0.49	2.44	.819
No/do not know	1.20	0.89	1.62	.238	0.95	0.61	1.47	.813	1.15	0.59	2.25	.685
Private insurance												
Yes	0.81	0.41	1.58	.530	1.47	0.39	5.52	.569	1.09	0.52	2.30	.821
No	1.16	0.97	1.38	.098	1.18	0.91	1.53	.200	1.16	0.90	1.50	.244
Do not know	1.00				1.00				1.00			
Smoking history												
Current smoker	1.22	0.89	1.66	.211	1.34	1.03	1.74	.029	1.25	0.99	1.58	.061
Past smoker	0.70	0.52	.93	.015	1.06	0.81	1.39	.653	1.01	0.78	1.30	.962
Non-smoker	1.00				1.00				1.00			
Alcohol consumption												
Never drink	1.00				1.00				1.00			
Less than 1 time per month	1.11	0.87	1.41	.410	0.72	0.57	0.93	.010	0.98	0.74	1.31	.904
1–4 times per month	1.10	0.89	1.36	.368	1.03	0.84	1.25	.802	0.96	0.76	1.22	.757
≥ 5 times per month	1.08	0.86	1.35	.498	0.89	0.70	1.12	.313	0.71	0.53	0.96	.024
Body mass index (BMI)												
Normal weight (18.5 ≤ BMI < 25)	1.00				1.00							
Under weight (BMI < 18.5)	0.97	0.83	1.13	.689	0.97	0.82	1.15	.734	0.90	0.74	1.10	.300
Obese (BMI ≥ 25)	0.93	0.60	1.45	.750	1.11	0.73	1.71	.618	1.22	0.91	1.65	.185
Exercise												
None	2.56	1.62	4.05	< .001	0.66	0.21	2.06	.473	2.03	0.23	17.92	.523
Mild	1.38	1.13	1.67	.001	1.12	0.87	1.45	.385	1.79	1.23	2.59	.002
High	1.00				1.00				1.00			
Self-rated health												
Very poor	3.32	1.63	6.75	.001	4.40	2.29	8.48	< .001	4.77	2.75	8.25	< .001
Poor	2.90	1.45	5.80	.003	2.85	1.62	5.04	< .001	2.47	1.06	5.78	.036
Fair	2.35	1.18	4.71	.016	2.06	1.20	3.54	.009	2.38	1.42	3.99	.001
Good	1.82	0.88	3.77	.108	1.36	0.76	2.40	.297	1.44	0.86	2.41	.168
Very good	1.00				1.00				1.00			
Stress level												
High	2.75	1.36	5.56	.005	1.62	0.41	6.46	.492	2.97	0.44	19.86	.261
Moderate	1.90	0.94	3.85	.073	1.04	0.26	4.18	.954	2.07	0.31	13.90	.454
Low	1.53	0.74	3.15	.246	0.64	0.16	2.62	.532	1.67	0.24	11.70	.604
None	1.00				1.00				1.00			
Pain												
None	1.00				1.00				1.00			
Mild	1.95	1.60	2.39	< .001	1.94	1.09	3.46	.024	1.69	0.52	5.43	.382
Severe	2.17	1.60	2.96	< .001	2.15	1.79	2.58	< .001	2.04	1.65	2.51	< .001
Depression												
Light	1.00				1.00				1.00			
Moderate	1.45	0.97	2.19	.072	2.99	1.33	6.74	.008	2.03	0.63	6.57	.236
Severe	1.63	1.34	1.98	< .001	1.28	1.01	1.63	.043	1.57	1.22	2.01	< .001

Logistic regression analysis with complex sampling design was performed by adjusting for covariates

Model 1 was adjusted by sex, age, marital status, family number and education level

Model 2 was adjusted by Model 1 as well as region, economic activity, income, occupation, medical insurance type and private insurance

Model 3 was adjusted by Model 2 as well as smoking, drink, body mass index, exercise, self-rated health status, stress level, pain and depression

*NHI* National Health Insurance, *Q* quartile, *OR* odds ratio, *CI* confidence interval, *OR* 95%, *CI* 95%

The factors affecting unmet healthcare needs differed by age groups. Education was the only significant factor in the younger age group (19–39 years). Individuals who received less than an elementary school education experienced more unmet healthcare needs compared with individuals who had college or higher education degrees (elementary school or less: OR 1.74, 95% CI 1.13–2.67). Furthermore, the high exercise group experienced more unmet healthcare needs than did their counterparts (none: OR 2.56, 95% CI 1.62–4.05; mild: OR 1.38, 95% CI 1.13–1.67), and there were more unmet healthcare needs with increased stress (high: OR 2.75).

Some factors were only significant in the group aged 40–59 years, who are economic activity ishigh. Compared to the white-collar group, the pink and the blue-collar groups with more physical activity experienced more unmet healthcare needs (pink collar: OR 1.88; blue collar: OR 1.36). Smokers experienced more unmet healthcare needs compared to the non-smokers (current smokers: OR 1.34). Concerning marital status, the married-no cohabitation, divorced, or bereaved group experienced more unmet healthcare needs compared to the unmarried group (married-no cohabitation, bereaved, or divorced: OR 1.71) In particular, individuals with lower income from the older group showed a clear tendency to experience more unmet healthcare needs (1Q (lowest): OR 1.30/ 2Q: OR 1.31/ 3Q: OR 1.44). Regardless of age, all groups showed a tendency to have more unmet healthcare needs with a worse subjective health state, worse pain, and a worse degree of depression.

## Discussion

This study analyzed the determinants of unmet healthcare needs among South Korean adults using KNHANES data for 2013–2017. In 2017, 9.5% of the sample experienced unmet healthcare needs. This percentage was 12.5% in 2013, which indicates that there has been an overall decline in unmet healthcare needs (see Additional files 1 and 2). This decline indicates the efficiency of the policies (such as reinforcement NHI coverage and an out-of-pocket limit) that have been implemented in South Korea in an attempt to reduce medical expenses [30, 31]. Previous studies have indicated that most of the reasons for unmet healthcare needs were economic-related; however, the recent data from 2017 showed that other reasons surpassed the economic reasons. One such determinant can be found based on the results of a recent domestic study, which reported that “time constraints” are the primary reason for unmet healthcare needs [10]. In our study, we showed that unsatisfactory medical care has significantly increased since 2013 because of time reasons rather than economic reasons (Table 1). This suggests that determinants besides economic factors should be considered to resolve unmet healthcare needs. However, it is important to focus not only on financial barriers, as the traditional policies have done, but also on other barriers. Based on our findings, we make the following three policy proposals.

### Improvement of policies concerning predisposing factors, particularly for women and younger age groups

We found that, compared to men, women experienced more unmet healthcare needs. Many women, especially mothers, feel that there are multiple barriers to their personal healthcare because they play a dual role, comprising responsibilities at work and at home, which impairs their ability to care for themselves [32]. Other studies have reported that women have traditionally been unable to obtain timely medical care because of their role as family “caretakers” [33]. Women in South Korean culture in particular, which is influenced by Confucian patriarchal values, tend to prioritize the medical needs of other family members over their own [34], and older women have been reported to have higher unmet healthcare needs as compared to younger women [14]. Moreover, compared to men, women may be more likely to experience a financial burden as a result of their lower social status, which causes restrictions on their social participation [35] and low health-related literacy [36, 37]. Due to this, women earn less and are financially dependent on their spouses.

Our results also showed that the younger group had greater odds of experiencing unmet healthcare needs than their older counterparts. There was a significant increase in use and access reasons as age increased. Previous studies reported that younger adults experienced less use- and access-related unmet healthcare needs than older adults, who experience relatively more health problems, regardless of sex [38, 39]. This can be interpreted as indicating that younger individuals more actively search for the medical services they require [40], have higher expectations regarding the quality of their healthcare, and have a greater likelihood of complaining when they are not satisfied with their health services [26, 41, 42].

### Policies that focus on the enabling factors, specifically low socioeconomic status, should be improved

Our results demonstrated that unemployment, low income, and blue-collar jobs (which involve heavy labor) are more likely to result in unmet healthcare needs (Table 4). According to an OECD report, people with low socioeconomic status are less likely to seek medical services they require [43]; this tendency is not specific to South Korea [11, 18, 44]. Economic status in particular is a major factor determining the use of medical services [45], and several countries have proposed multiple policies to address financial barriers in an effort to ensure the use of essential medical services [46–49]. In South Korea, financial barriers to healthcare remain despite the country’s universal health insurance system [50, 51]. Notably, however, prior findings have led to the implementation of improved policies that focus on access, which resulted in an expansion of the coverage of the NHI in South Korea, consequently reducing the costs of medical services for people with low socioeconomic status [52–55].

### Addressing need factors, pain, poor subjective health status, and depression because they are key determinants of unmet healthcare needs

Our findings show that the lowest subjective health status and high levels of stress, pain, and depression are significantly associated with unmet healthcare needs. These results are consistent with those of the previous studies; that is, poor subjective health status [56], increases pain [57], and high stress and depression [58, 59] cause more unmet healthcare needs. In particular, participants with poor subjective health status were in serious need of medical services. Therefore, acceptability-related reasons for unmet healthcare needs may have a strong influence on such individuals’ access to medical services [21]. Moreover, severe depression may have a significant impact on access-related reasons for unmet healthcare needs, as depression can lead to poor health behavior [60] and financial burdens [61, 62]. Further, the associations between obesity and low accessibility were discovered: they were found to be related to the physical restrictions owing to obesity-associated pain and physical discomfort. A previous study on the association between obesity and unmet healthcare needs reported that obese older adults are more likely to experience unmet physical activity [63].

Based on these results and those of previous studies, women who are young, have no or a low level of education, are unemployed or employed in blue-collar jobs, and who are severely depressed are more vulnerable and more likely to have unmet healthcare needs as compared to their counterparts. Thus, less-privileged populations with low socioeconomic status require more medical attention and experience diverse health problems [64].

This study had some limitations. First, self-report data were used to measure unmet healthcare needs; therefore, the overall reliability of the data may be questionable [65]. Additionally, the association between various factors and unmet healthcare needs may have been under- or over-reported. However, this would not restrict the generalization of the results; previous studies have suggested that self-reported evaluation of unmet healthcare needs is an appropriate method of analyzing population-level national surveys [5]. Second, the KNHANES provides secondary data, which limited our ability to conduct a detailed analysis of the risk factors. The types of medical institutes (e.g., hospitals and clinics), the specific diseases, the regions, and types of services for which patients encountered unmet healthcare needs should be further analyzed [23]. Finally, we analyzed five-year data, from 2013 to 2017. A cross-sectional study design was used instead of a longitudinal study design, as each individual participated only once in the survey over the five-year period. Therefore, our results, which reflect individual trends, should be supplemented by accumulated longitudinal data [50].

Despite these limitations, our research is significant because it provides up-to-date information concerning unmet healthcare needs, utilizing the KNHANES 2017—the latest reliable data for South Korea. One particular strength of this study lies in the classification of the causes of unmet healthcare needs. Unmet healthcare needs are widely used indicators for evaluating a country’s healthcare system. Therefore, our findings may be a good reference for countries that have similar healthcare systems to that of South Korea, such as France, Germany, Japan, and Ireland, where public and private insurance systems share the burden of medical expenses [66].

## Conclusions

Although South Korea has witnessed a steady decrease in unmet healthcare needs, we found that 9.5% of the participants continue to experience these barriers to adequate healthcare. Women with low socioeconomic status experienced the highest level of unmet healthcare needs. Therefore, we recommend the implementation of policies that reduce unmet healthcare needs by enhancing the healthcare system at the national-level and targeting specific groups.

## Supplementary Information

**Additional file 1:** Percentage of population reporting unmet healthcare needs by year.

**Additional file 2:** Trend of population reporting unmet healthcare needs by year.

## Data Availability

All original data are publicly available free of charge from the KNHANES website (http://knhanes.cdc.go.kr) for the purposes of academic research.

## Abbreviations

CHS: Community Health Survey
CI: Confidence interval
KCDC: Korea Center for Disease Control and Prevention
KNHANES: Korea National Health and Nutrition Examination Survey
NHI: National Health Insurance
OECD: Organization for Economic Cooperation and Development
OR: Odds ratio
SE: Standard error
WHO: World Health Organization
