# Supplementary material for: Determining the reasons for unmet healthcare needs in South Korea: a secondary data analysis
Source: Health Qual Life Outcomes. 2021 Mar 20;19:99. doi: 10.1186/s12955-021-01737-5 (PMC7981839; doi:10.1186/s12955-021-01737-5)
Supplement: Supplementary file 1 — Additional file 1: Percentage of population reporting unmet healthcare needs by year. [file 12955_2021_1737_MOESM1_ESM.docx]

Additional file 1. Percentage of population reporting unmet healthcare needs by year.

| Type | Unmet | 2013 | | 2014 | | 2015 | | 2016 | | 2017 | |
| --- | --- | --- | --- | --- | --- | --- | --- | --- | --- | --- | --- |
|  |  | N | (%) | N | (%) | N | (%) | N | (%) | N | (%) |
|  | No | 4596 | (86.9) | 4351 | (87.8) | 4384 | (87.2) | 5139 | (90.8) | 5136 | (90.6) |
| Unweighted | Yes | 695 | (13.1) | 605 | (12.2) | 642 | (12.8) | 519 | (9.2) | 531 | (9.4) |
|  | Total | 5291 | (100.0) | 4956 | (100.0) | 5026 | (100.0) | 5658 | (100.0) | 5667 | (100.0) |
|  | No | 6384370 | (87.6) | 6235757 | (88.1) | 6354086 | (87.3) | 7067622 | (91.3) | 7041400 | (90.5) |
| Weighted | Yes | 902254 | (12.4) | 839296 | (11.9) | 920566 | (12.7) | 671091 | (8.7) | 742663 | (9.5) |
|  | Total | 7286624 | (100.0) | 7075053 | (100.0) | 7274652 | (100.0) | 7738713 | (100.0) | 7784063 | (100.0) |
