# Supplementary figures and images for: Determining the reasons for unmet healthcare needs in South Korea: a secondary data analysis
Source: Health Qual Life Outcomes. 2021 Mar 20;19:99. doi: 10.1186/s12955-021-01737-5 (PMC7981839; doi:10.1186/s12955-021-01737-5)

Additional file 2. Trend of population reporting unmet healthcare needs by year.


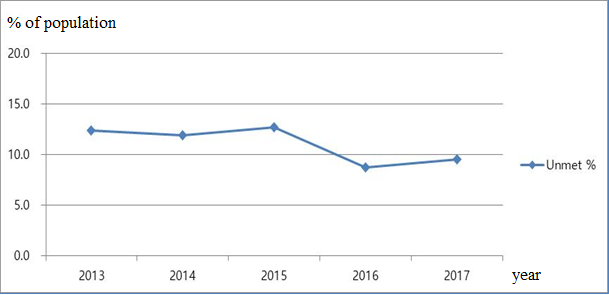

Supplement: Supplementary file 2 — Additional file 2: Trend of population reporting unmet healthcare needs by year. [file 12955_2021_1737_MOESM2_ESM.docx]
